# The efficacy of gradual reduction using two-stage traction for developmental dysplasia of the hip in southern China

**DOI:** 10.3389/fped.2024.1335490

**Published:** 2024-03-11

**Authors:** Hui Chen, Xiang-xuan Wang, Zhao Chen, Yihua Ge

**Affiliations:** ^1^ Department of Pediatric Orthopedics, Fujian Maternity and Child Health Hospital College of Clinical Medicine for Obstetrics & Gynecology and Pediatrics, Fujian Medical University, Fuzhou, China; ^2^ Department of Pediatric Orthopedics, Fujian Children’s Hospital (Fujian Branch of Shanghai Children’s Medical Center), Fuzhou, China; ^3^ Department of Orthopaedics, Shanghai Children’s Medical Center, Shanghai Jiao Tong University School of Medicine, Shanghai, China

**Keywords:** developmental dysplasia of the hip, traction, closed reduction, arthrography, efficacy

## Abstract

**Purpose:**

This study aimed to report the preliminary outcome of gradual reduction (GR) utilizing two-stage traction (TST) compared with traditional traction (TT) in the treatment of developmental dysplasia of the hip (DDH) and to evaluate whether the prognosis of the TST is better than that of TT.

**Methods:**

The following information on children diagnosed with DDH who underwent treatment with GR using two-stage traction or traditional traction between June 2016 and August 2017 was collected: sex, age, weight, acetabular index (AI), International Hip Dysplasia Institute (IHDI) classification, femoral head ossification, traction time, reduction quality, and labrum shape in arthrography. The AI, IHDI classification, second operation rate, and incidence of femoral head avascular necrosis (AVN) were analyzed after the final comprehensive 1-year follow-up.

**Results:**

In this study, 27 cases (31 hips: 18 left and 13 right) were enrolled, with 18 hips (16 cases) assigned to the TT group and 13 hips (11 cases) assigned to the TST group, with the corresponding average age at diagnosis of 5.56 ± 1.66 and 4.06 ± 1 months (*p* < 0.001). For both TT and TST groups, the average age at operation was 6.01 ± 1.67 and 65 ± 0.86 months (*p* = 0.435), the distribution of affected left and right sides was 10/8 and 8/5 hips (*p* = 1), and the average initial AI was 37.11 ± 3.26 and 36.77 ± 4.34 (*p* = 0.804), respectively. IHDI classification III/IV was observed in 15/3 and 11/2 hips, respectively (*p* = 1). Femoral head ossification was present in 6/18 hips in the TT group and 2/13 hips in the TT group (*p* = 0.412). The total traction time was 13.22 ± 2.6 days for the TT group and 49.23 ± 25.77 days for the TST group (*p* < 0.001). After GR, IHDI classification III/IV was observed in 9/9 and 12/1 hips, respectively (*p* = 0.02). AVN was present in 5/18 hips in the TT group and 0/13 hips in the TST group (*p* = 0.048), while the need for a second operation was approved in 5/18 hips in the TT group and 1/13 hips in the TST group (*p* = 0.359) at the final follow-up.

**Conclusions:**

Two-stage traction can significantly decrease the ratios of IHDI classifications III and IV and the incidence of AVN compared to traditional traction; also, it significantly reduces the total traction time.

## 1 Introduction

Developmental dysplasia of the hip (DDH) includes a series of deformities ranging from mild dysplasia of the hip to total dislocation of the hip. The primary goal of DDH treatment is to achieve and maintain a definite concentric reduction of the femoral head and the acetabulum, thereby promoting proper development of the acetabulum. Another important treatment goal is to minimize iatrogenic avascular necrosis (AVN) of the femoral head (1).

The established treatment strategy for DDH in patients younger than 18 months involves obtaining a stable concentric reduction by open or closed methods under sedation or anesthesia and to maintain reduction with a spica cast (1, 2). Whether traction should be performed before reduction remains controversial (1, 3–10). Studies report that vertical traction has a high rate of reduction and a low incidence of femoral head necrosis (3, 4). However, the long hospitalization period is a major factor restricting the application of traction. Although traction can produce satisfactory long-term clinical results, its feasibility has been limited in today’s fast-paced medical environment due to the prolonged hospital stay it necessitates.

In this study, we utilized a two-stage traction (TST) gradual reduction protocol, which consists of at-home horizontal traction and in-hospital vertical traction, to improve the success rate of reduction and minimize the risk of AVN without extending the length of hospital stay.

## 2 Materials and methods

### 2.1 Patients

**Figure 1:**
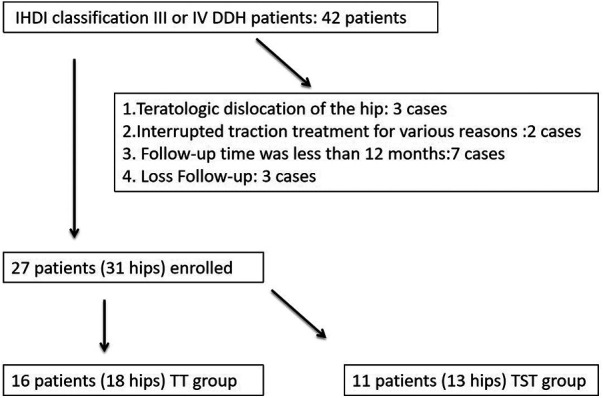
Flow diagram detailing the methods used to identify the study.

The information on children diagnosed with DDH and treated at Shanghai Children's Medical Center, affiliated with Shanghai Jiao Tong University and Fujian Children's Hospital, between June 2016 and August 2021 was collected. Files of all the patients were reviewed after approval from the hospitals. The inclusion criteria included the following: (1) age < 12 months; (2) diagnosis of idiopathic DDH; (3) successful completion of the two-stage traction reduction treatment; (4) complete radiographic data; and (5) follow-up duration of more than 12 months. The exclusion criteria were as follows: (1) teratologic dislocation of the hip (three cases); (2) interrupted traction treatment for various reasons (two cases); (3) follow-up time duration of less than 12 months (seven cases); and (4) cases that lacked any follow-up (three cases) (Figure 1).

The traction was performed by YG or another doctor under his supervision; YG conducted ultrasound examinations and interpreted radiographs at the hospital. However, traction at home was installed by parents trained by YG, and radiographic examinations were performed in our hospital once a week without an ultrasound examination.

The patients were categorized into either the traditional traction (TT) group or the TST group; indications for TT or TST were an International Hip Dysplasia Institute (IHDI) classification of III or IV and failure to achieve a reduction in the dislocation after 3 weeks of Pavlik treatment.

### 2.2 TT group

Similar to the protocol of Fukiage et al. (3), the traditional traction stage involved ultrasound-guided progressive abduction vertical traction performed in the hospital. The hip was initially flexed at 100° and abducted at 10°, with a 1.5-kg load for each leg. Abduction was raised to 60° over the course of 1 week. An anterior axial ultrasound view was utilized to evaluate the hip dislocation every 2–3 days to monitor the reduction progress of the femoral head; another 0.5 kg of traction load would be added if the hip remained dislocated. Padding under the greater trochanter was also used to assist in the reduction of the femoral head. Once the hip was reduced to the acetabulum on the anterior axial view ultrasound, the weight of traction was reduced by 0.5 kg per day to promote the gradually “docking” of the femoral head into a concentric position.

### 2.3 TST reduction group

**Figure 2:**
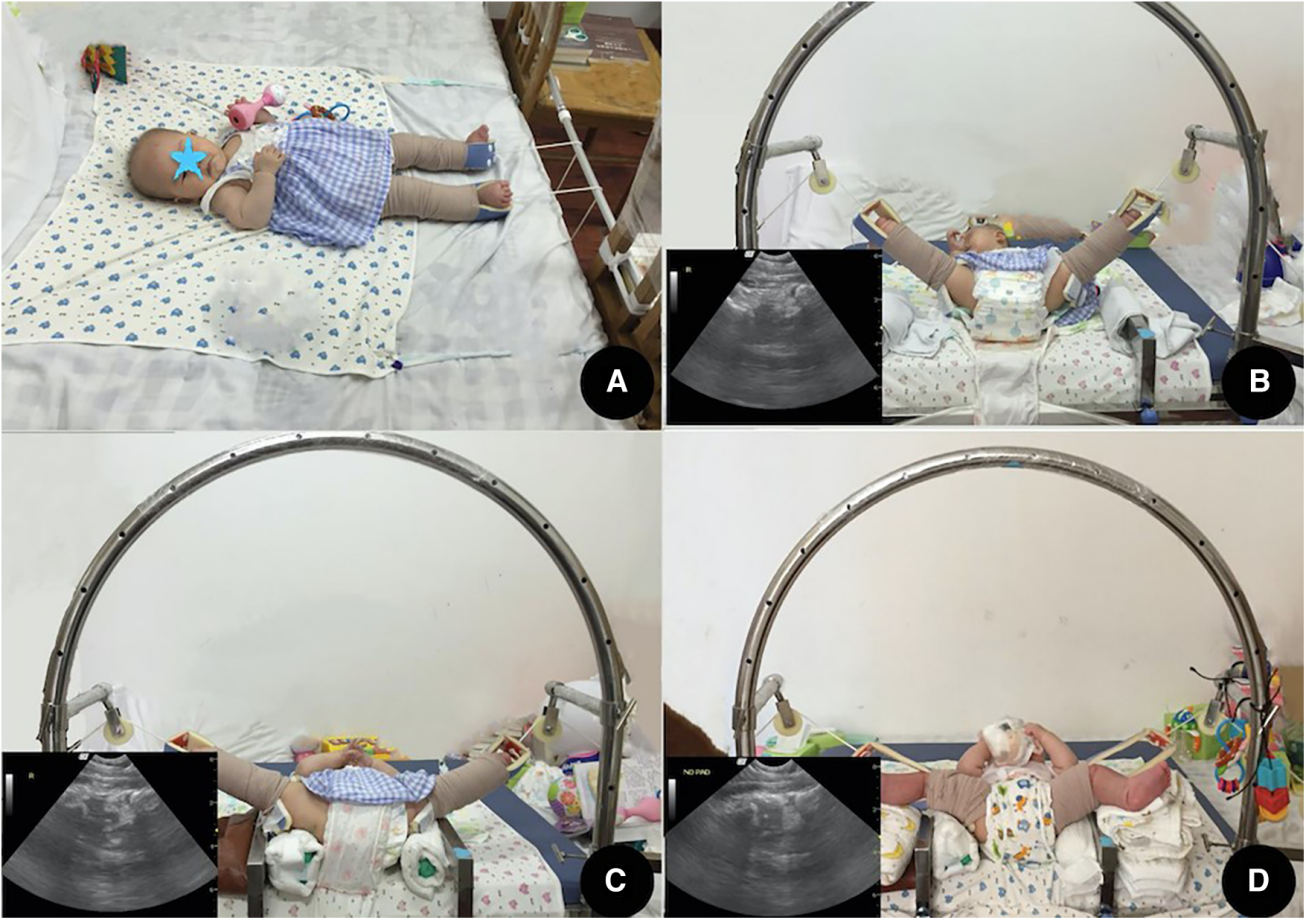
Two-stage traction. Stage 1: (**A**) horizontal traction at home. Stage 2: (**B**–**D**) ultrasound-guided vertical traction in the hospital. (**B**) Day 4, abduction was increased to 50°, and anterior hip ultrasound viewed the posterior dislocated femoral head. (**C**) Day 7, after using padding and adding the traction load, the femoral head was reduced to the acetabulum, but some distance between the femoral head and the acetabulum can be seen on the anterior hip ultrasound. (**D**) Day 12, after reducing the traction load, the femoral head gradually “docked” to a concentric position. The minimized distance between the femoral head and the acetabulum can be seen on the anterior hip ultrasound).

Our TST strategy involves a longer traction preparation period than TT. The patient is immobilized in a jacket attached to the mattress, and skin horizontal traction with an initial load of 1.0 kg for each leg is applied at home during the preparation stage. The patient's family is instructed to install the traction device at home. A pelvic radiograph is performed every week to monitor the descent of the femoral head. The vertical distance between the midpoint of the proximal femoral physis and the Hilgenreiner line, or “distance A,” is utilized to evaluate the progress of horizontal traction on the pelvic radiograph (4). Another 0.5 kg of traction load is added if the femoral head does not descend after a week. The descent is defined as sufficient when the midpoint of the proximal femoral physis descended to 8 mm below the level of the Hilgenreiner line, and the gradual reduction stage is then applied (3). After the descent, they are transferred to the second stage of traditional traction as above (Figure 2).

### 2.4 Arthrographic and spica casts

**Figure 3:**
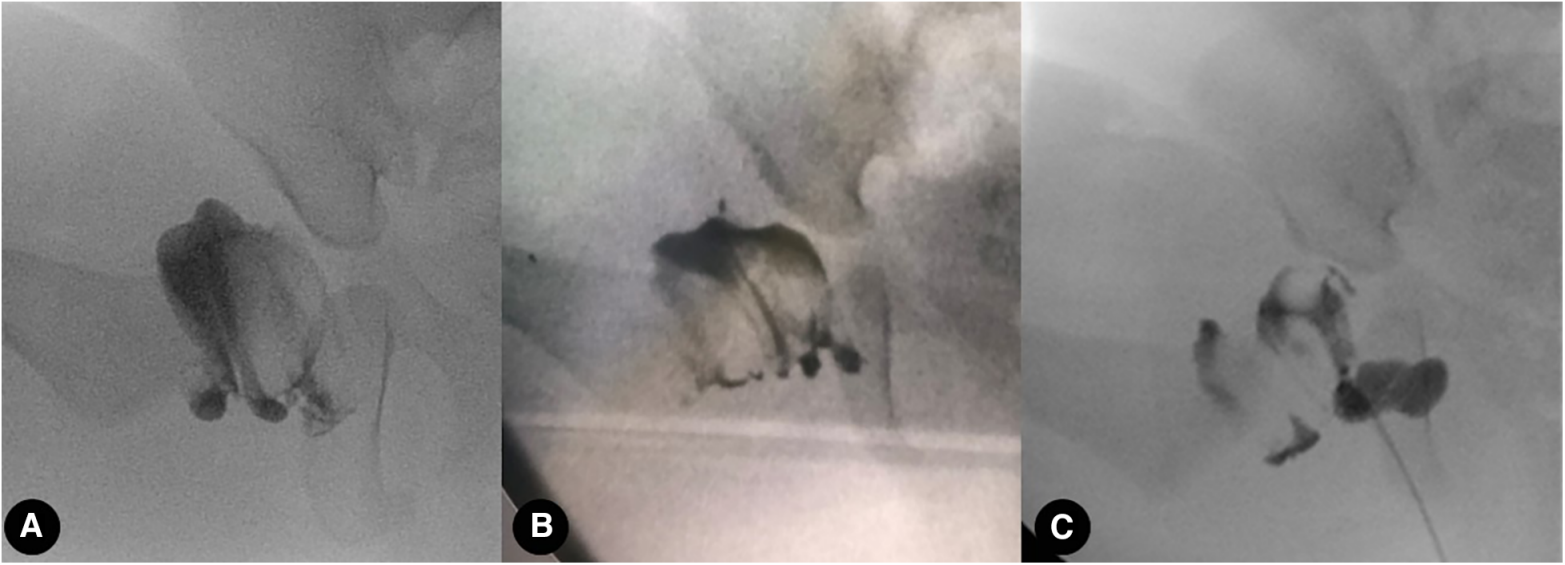
Arthrography under general anesthesia was performed to evaluate the quality of gradual reduction. (**A**–**C**) Tonnis grade 1, 2, and 3 reduction quality of the hip, respectively.

To maintain reduction, arthrography under general anesthesia was performed to evaluate the quality of gradual reduction and to decide whether an extra open reduction was required (Figure 3) in both groups according to the arthrographic grades of reduction (Tonnis) (11), according to the Tonnis criteria for arthrography. The Tonnis criteria are described as follows: Grade 1, the femoral head is fully reduced and is well approximated to the ischial part of the acetabulum (Kohler's teardrop); Grade 2, the femoral head is below the labrum but is somewhat lateralized due to constriction of the capsule and encroachment of the superior and inferior labrum and transverse ligament; Grade 3, the femoral head is not below the labrum and is outside the acetabulum, the labrum is inverted, or there is marked constriction of the capsule. The hip was then placed in a spica cast (hip flexion 100°–110°, abduction less than 60°) for 3 months, after which an abduction brace (similar to a spica cast but covering a shorter portion of the leg above the knee) was put on for 6–12 months after cast removal in both groups.

### 2.5 Observation indicators

**Figure 4:**
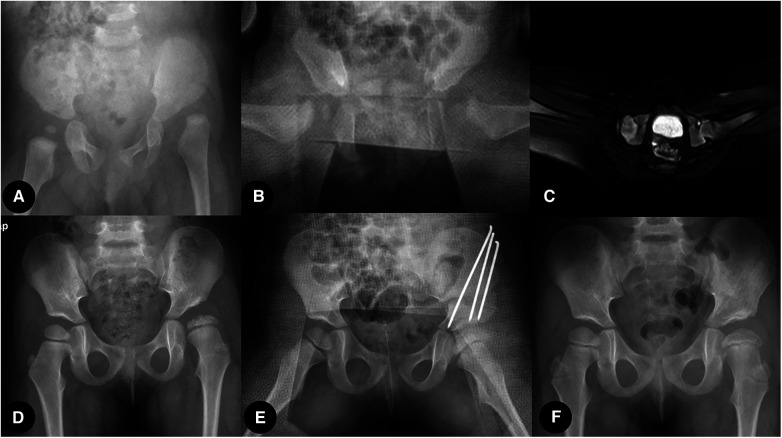
Traditional overhead traction group case: (**A**) Six-month-old girl patient with left hip dislocation. (**B**,**C**) X-ray film and MRI transaction image showing successful close reduction after 2 weeks of overhead traction. (**D**) Residual acetabulum dysplasia, subluxation, and AVN of the left femoral head were confirmed at 2.5 years after close reduction. (**E**) Open reduction and Salter osteotomy were performed. (**F**) One year after the second surgery, the reduction was maintained, and the acetabulum was remodeled.

**Figure 5:**
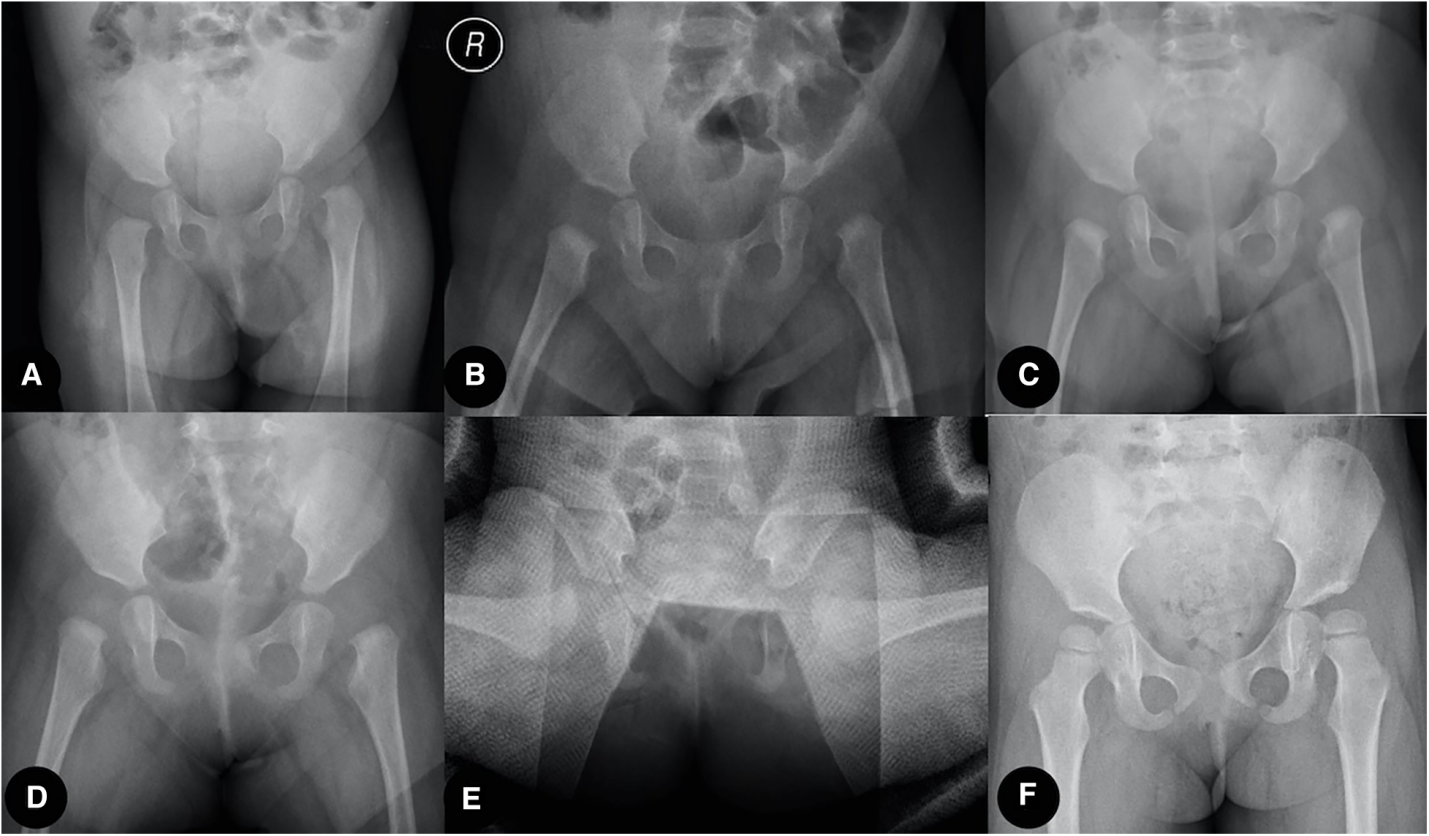
Two-stage traction reduction group case: (**A**) Five-month-old girl patient with left hip dislocation. (**B**–**D**) At-home horizontal traction achieved a sufficient proximal femoral physis descent, and the ultrasound-guided in-hospital vertical traction was applied. (**E**) After being evaluated by arthrography, the hip was immobilized in a spica cast for 3 months. (**F**) Reduction was maintained at the final follow-up.

Sex (M/F), age, weight, acetabular index (AI), IHDI classification, femoral head ossification, total traction time, traction time of each stage, Tonnis arthrography classification, and the shape of the labrum in arthrography were evaluated. The prevalence of femoral head AVN was also recorded and analyzed at a 1-year follow-up. One case accepted traditional overhead traction, and the other accepted two-stage traction, as shown in Figures 4, 5.

## 3 Results

A total of 31 hips (27 cases) were enrolled in this study. The mean follow-up time was 26.55 ± 11.5 months. A total of 18 hips (16 cases) were assigned to the TT group, with the average age at diagnosis of 5.56 ± 1.66 months and the average age at operation of 6.01 ± 1.67. Among these, 10 hips were affected on the left side and 8 hips were affected on the right side. The average initial AI was 37.11 ± 3.26, with IHDI classification indicating 15 hips as grade III and 3 hips as grade IV. Femoral head ossification was present in 6 hips and absent in 12 hips. The other 13 hips (11 cases) were assigned to the TST group, with the average age at diagnosis of 4.06 ± 1 months and the average age at operation of 5.65 ± 0.86. Among these, eight hips were affected on the left side and five hips were affected on the right side. The average initial AI was 36.77 ± 4.34. The IHDI classification was III in 11 hips and IV in 2 hips, and femoral head ossification was present in 2 hips and absent in 11 hips. The difference in age at diagnosis was significantly lower in the TST group than in the TT group, but there were no significant differences in gender, operation age, affected side, AI, IHDI classification, and femoral head ossification (Table 1).

**Table 1:** Data of patients of traditional and two-stage traction groups.

Group	Traditional group (*n* = 18 hips)	Two-stage group (*n* = 13 hips)	*p*-value
Gender (male/female)	7/11	2/11	0.237
Age at diagnosis	5.56 ± 1.66	4.06 ± 1	<0.001
Age at operation	6.01 ± 1.67	5.65 ± 0.86	0.435
Side (left/right)	10/8	8/5	1.000
Initial AI	37.11 ± 3.26	36.77 ± 4.34	0.804
IHDI classification			1.000
III	15	11	
IV	3	2	
Femoral head ossification			0.412
Yes	6	2	
No	12	11	
Total traction time (days)	13.22 ± 2.6	49.23 ± 25.77	<0.001
Horizontal traction time	0	35.38 ± 25.70	
Vertical traction time	13.22 ± 2.6	13.85 ± 1.72	0.458

The total traction time was 13.22 ± 2.6 days (ranging from 7 to 19 days), which was equal to the hospitalized vertical traction time in the TT group. However, the total traction was 49.23 ± 25.77 days (ranging from 28 to 105 days) in the TST group, which included horizontal traction time at home and vertical traction time in the hospital, with an average of 35.38 ± 25.70 days (ranging from 14 to 91 days) and 13.85 ± 1.72 days (ranging from 9 to 17 days), respectively. The total traction time in the TT group was significantly lower than in the TST group (*p* < 0.001), but the vertical traction time in the hospital was similar between the two groups (*p* = 0.458) (Table 1).

Intraoperative arthrography was performed on all patients. Tonnis classification revealed 12 hips in grade 1, 4 hips in grade 2, and 2 hips in grade 3 in the TT group, while 8 hips in grade 1, 3 hips in grade 2, and 2 hips in grade 3 in the TST group. Hips with Tonnis grades 1 or 2 received instant spica cast fixation after arthrography, while hips with Tonnis grade 3 underwent open reduction via an anterolateral approach in both groups. All hips were successfully reduced, and no re-dislocation was observed at the final follow-up in both groups (Table 2).

**Table 2:** Preliminary outcomes of patients of traditional and two-stage traction groups.

Group	Traditional group (*n* = 18 hips)	Two-stage group (*n* = 13 hips)	*p*-value
Tonnis arthrographic grade			
I	12	8	0.741
II	4	3	
III	2	2	
AI	25.06 ± 5.86	23.52 ± 4.31	0.429
IHDI classification			0.02
I	9	12	
II	9	1	
AVN			0.048
Yes	5	0	
No	13	13	
Second operation			0.359
Yes	5	1	
No	13	12	

At the time of the final follow-up, the average AI was 25.06 ± 5.86 in the TT group and 23.52 ± 4.31 in the TST group; the difference was not significant (*p* = 0.429). The IHDI classification revealed 9 hips in grade I and 9 hips in grade II in the TT group, while 12 hips in grade I and 1 hip in grade II in the TT group; the difference was significant (*p* = 0.02). The TST group had no cases of AVN of the femoral head, whereas the TT group had five cases; the difference was significant (*p* = 0.048). All five cases of AVN were mild, with four cases classified as type I AVN and one case as type II AVN according to Kalamchi’s criterion (12). At the last follow-up, five hips in the TT group and one hip in the TST group received a second operation (salter pelvic osteotomy, varus and derotate femoral osteotomy were performed in all the six cases) due to residual deformity (*p* = 0.359) (Table 2).

## 4 Discussion

The therapeutic principle of DDH aims to achieve a stable concentric reduction and maintain this alignment until the hip matures while avoiding iatrogenic AVN of the femoral head (2, 13). One-time reduction, whether closed or open, may result in varying proportions and degrees of iatrogenic AVN in DDH patients with severe dislocation (1, 14). The occurrence of AVN can seriously affect the prognosis, which has been proven unavoidable (15). Fortunately, several studies on DDH treatment by gradual traction reduction have reported very low rates of AVN during long-term follow-up (3–9) in recent years. Although certain residual deformities necessitate a second surgery, the gradual traction reduction strategy has demonstrated excellent protection of the blood supply to the femoral head (5, 10) and has been effective in preventing iatrogenic AVN.

Compared to one-time reduction, preoperative traction before closed reduction is predicted to decompress the soft tissue around the hip joint, reduce pressure on the femoral head and acetabulum following reduction, improve the success rate of reduction, and reduce the risk of femoral head necrosis, especially applicable to patients who cannot be reduced during the Pavlik brace treatment process. However, the effectiveness of this method is subject to debate. Sucato et al. found that closed reduction following vertical traction did not improve the success rate of closed reduction alone or reduce the incidence of AVN (1). In contrast to traditional traction, a new traction strategy was reported by Kaneko et al. to achieve a gradual reduction of the femoral head (4), aiming to conserve the blood supply to the femoral head and avoid necrosis through delayed traction reduction. The entire process is monitored by x-ray and ultrasound with quantitative evaluation indicators, which can aid in preventing traction failure due to low compliance and inadequate monitoring. Japanese scholars (Fukiage et al. and Kaneko et al.) using this process have reported a 96%–99% success rate, which is significantly higher than previous studies employing traditional traction (Sucato et al., 60.9%) (1, 3, 4). We also speculate that it prevents the risk of AVN by preventing abrupt changes in the blood circulation in the femoral head caused by one-time closed reductions. In our study, there were no occurrences of AVN in the TST group at the 1-year follow-up, despite two hips remaining Tonnis grade 3 reduction quality following traction (which required an open reduction to complete the process). However, in the TT group, 5 out of 18 hips showed AVN, showing a significant difference (*p* = 0.048) compared to the TST group. Therefore, we postulate that this method could give the dislocated femoral head time to adapt to the new environment gradually created by the traction. We believe this slow change process could minimize the AVN rate and explain the low necrosis rate of the femoral head reported by Kaneko et al. (4).

According to the studies, gradual reduction treatment also has a certain proportion of residual deformity (19.2%–64%) (3, 4, 16, 17). With this method, we hoped to reduce the residual deformity rate by enhancing the reduction quality. In our patient cohort, every patient received arthrography under general anesthesia to evaluate the reduction quality. Only cases with concentric reduction (Tonnis grade 1) and an approximation of concentric reduction (Tonnis grade 2) were deemed acceptable. Patients with non-concentric reduction (Tonnis grade 3) received an anterolateral approach for open reduction to improve the reduction quality. As a result, 1/13 hips in the TST group had a residual deformity and required a second operation, but 5/18 hips in the TT group had residual deformities and received a second operation; however, this difference was not statistically significant (*p* = 0.359).

However, due to lengthy hospitalization, some children may become uncooperative with traction. These challenges may encourage families to opt for single-stage traditional traction before closed or open reduction. The modified gradual traction reduction using two-stage traction offers several advantages. Horizontal traction is performed at home during the preparation stage, which reduces the hospitalization time for vertical traction to approximately 2 weeks. At the same time, it provides good reduction quality and simultaneously protects the blood supply to the femoral head.

Our study had several limitations. First, it is constrained by its retrospective study design. Second, it has a small sample size and a short follow-up period. Given our small sample size and short follow-up time, we cannot conclusively establish a link between the decline in residual deformity rate and our efforts to improve the reduction quality. Larger sample sizes and long-term follow-ups are required to test the validity of this method.

## 5 Conclusion

Two-stage traction or traditional traction in DDH has similar clinical and radiological results, with no significant differences observed in the need for revision surgery between the groups. Compared to conventional traction, two-stage traction can significantly decrease the incidence of AVN and the ratios of IHDI classifications III and IV. The total traction time was significantly longer in the TST group than in the TT group; however, the traction time in the hospital was similar between the two groups, indicating that it is an appropriate early treatment strategy for DDH under the current medical model when the socio-economic conditions allow its implementation.

## Data Availability

The original contributions presented in the study are included in the article/Supplementary Material, further inquiries can be directed to the corresponding author.
